# Implementation of iodine biokinetic model for interpreting I-131 contamination in breast milk after the Fukushima nuclear disaster

**DOI:** 10.1038/srep12426

**Published:** 2015-07-22

**Authors:** Kotaro Tani, Osamu Kurihara, Eunjoo Kim, Satoshi Yoshida, Kazuo Sakai, Makoto Akashi

**Affiliations:** 1 National Institute of Radiological Sciences, 4-9-1, Anagawa, Inage-ku, Chiba city 263-8555 Japan

## Abstract

After the accident at the Fukushima Daiichi Nuclear Power Plant run by Tokyo Electric Power Company in 2011, breast milk samples obtained from volunteers living in Fukushima and neighboring prefectures were examined and small amounts of I-131 (2.2–36.3 Bq/kg) were detected in some samples. In this work, the I-131 concentrations in breast milk from nursing mothers in Ibaraki prefecture were calculated based on the iodine biokinetic model during lactation together with time-variable intake scenarios by inhalation of ambient air and ingestion of tap water, using the authors’ code. The calculated I-131 concentrations in breast milk generally agreed with those measured for the volunteers. Based on the results, thyroid equivalent doses to breast-fed infants were estimated for each place of residence of the volunteers on the assumption that these infants consumed 800 ml of breast milk every day, resulting in 10–11 mSv for Mito and Kasama cities and 1.1–1.8 mSv for Tsukuba and Moriya cities. It was suggested that breast milk consumption could be a major contributor to internal dose of breast-fed infants in areas with mild I-131 pollution; however, further studies considering personal behavior surveys would be necessary to estimate individual doses.

The Fukushima Daiichi Nuclear Power Plant (FDNPP) run by Tokyo Electric Power Company experienced an unprecedented nuclear accident due to the failure of cooling functions for reactor cores amid the tremendous tsunamis generated by the Great East Japan Earthquake on March 11, 2011. Significant amounts of radionuclides (I-131: 120–150 PBq, Cs-137: 10–30 PBq) were released into the environment^1^,^2^,^3^, causing radiation exposure to the public. Understanding the resulting exposure doses to residents of the affected areas by the accident is critical for clarifying the longer-term potential health risks. Various attempts of dose reconstruction have been made^4^,^5^, but internal doses received from short-lived radionuclides still remain highly uncertain because of insufficient information, and especially from the limited number of human measurements^6^,^7^.

It has been demonstrated in relation to the Chernobyl nuclear accident that increased thyroid cancers among children are linked to thyroid exposure mainly from the intake of foodstuffs contaminated with I-131^8^,^9^,^10^,^11^. In this context and amid widespread concern about mothers with infants, breast milk samples were collected from nursing mothers in regions neighboring Fukushima. According to Unno *et al.*^12^, small amounts of I-131 were occasionally detected in samples from nursing mothers living in Fukushima, Chiba and Ibaraki prefectures, suggesting that mild I-131 pollution in these areas might have caused incorporation of I-131 by nursing mothers, resulting in I-131 contamination in secreted breast milk. However, the intake routes by nursing mothers have not been explained so far. Therefore, the main focus of this study was whether fundamental intake pathways for nursing mothers based on environmental data gave a reasonable explanation for the observed levels of I-131 concentrations in breast milk.

## Results

**Figure 1:**
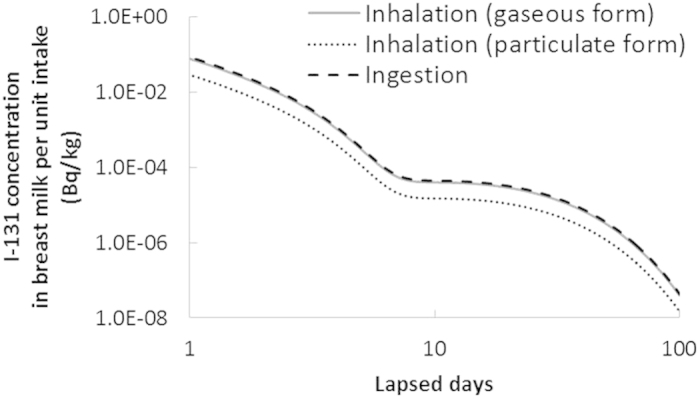
Model prediction of I-131 concentration in breast milk after acute intake.

The iodine biokinetic model during lactation for interpreting the observed I-131 contamination in breast milk was implemented using a code developed by the authors^13^. The model prediction of I-131 concentration in breast milk after acute intake is illustrated in Fig. 1. The results shown in the figure accurately reflected the data of publication 95 by the International Commission on Radiological Protection (ICRP)^14^ in terms of the fraction secreted into breast milk. The concentration in breast milk in case of particulate form inhalation is lower than that in case of gaseous form inhalation or ingestion, as only 34% of the intake amount is absorbed by blood through particulate form inhalation, while most of it is absorbed through gaseous form inhalation or ingestion^15^,^16^.

**Figure 2:**
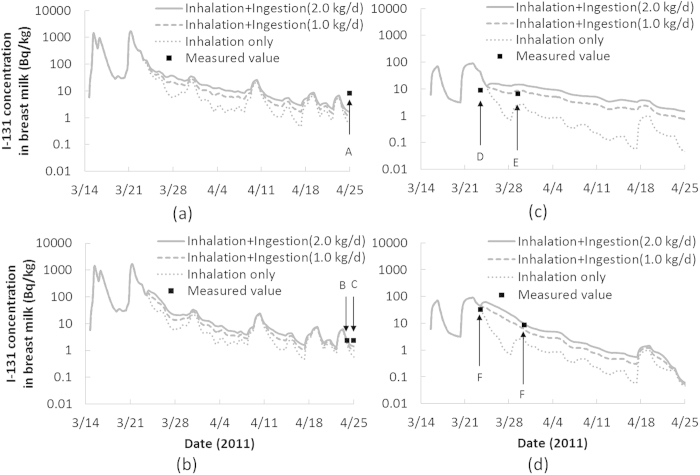
Calculated and observed I-131 concentration in breast milk: (**a**) Person A (Mito city), (**b**) Persons B and C (Kasama city), (**c**) Persons D and E (Tsukuba city) and (**d**) Person F (Moriya city).

The calculated I-131 concentrations in breast milk based on the intake scenarios by the combination of ambient air inhalation and tap water ingestion are displayed in Fig. 2. The measured data for Persons A-F are also shown in the figure for comparison with those calculated for their respective cities of residence. The measured and calculated concentrations in the figure are also shown in Table 1. The range of each calculated value denotes the margins of the maximum and minimum calculated concentrations on each date of sampling, taking into account both daily variation and various intake scenarios, with 0.0–2.0 (kg/d) for daily consumption of tap water and 13.1–18.4 (m^3^/d) for daily effective breathing volume (as described in Methods). Although the precise sampling time during the day was unknown and the measured values were corrected at the time when each measurement was started, these have negligible influence on the above margins. As a whole, the measured and calculated I-131 concentrations agreed with each other well enough to explain the fact of the contamination in breast milk.

## Discussion

We performed a straightforward procedure for estimating the intake amount based on individual monitoring data coupled with an assumed intake scenario to determine the fraction of retention and excretion of the radionuclides incorporated as a function of time. For example, based on the result shown in Fig. 1, the I-131 intake amount for Person A, who lived in Mito city, was calculated as 1.1 MBq, assuming an acute intake scenario by inhalation of the gaseous form on 15 March 2011. However, this amount was larger by two orders of magnitude than the total I-131 activity according to intake scenarios based on environmental data of air and tap water before the measurement of breast milk. Therefore, acute intake scenarios shortly after the accident, which have often been used in internal dose estimations for Fukushima residents^6^,^17^,^18^, seem to be quite inappropriate in this case.

Estimated I-131 intake amounts and thyroid equivalent doses to nursing mothers and their breast-fed infants are shown in Table 2. The intake amounts of nursing mothers were obtained by the assumed intake scenarios described in Methods. As for the breast-fed infants, those by inhalation were obtained using parameters related to the breathing volume and time budget for 3-month-old infants: 0.09 (m^3^/h) and 17 (h) for asleep time and 0.19 (m^3^/h) and 7 (h) for awake time^16^. In addition, the doses by ingestion of infants who supposedly consumed 800 ml^14^ of breast milk every day from March 14 to April 25 in 2011 were obtained based on the results shown in Fig. 2. Thyroid equivalent doses were calculated by the dose coefficients of adults and 3-month-old infants recommended by ICRP^16^,^19^. The resulting thyroid equivalent doses through inhalation of gas and particulates to nursing mothers were higher than those through ingestion, especially for Mito and Kasama cities. In contrast, the doses through inhalation to breast-fed infants were lower than those through ingestion via breast milk. The doses by ingestion were 10–11 mSv for Mito and Kasama cities and 1.1–1.8 mSv for Tsukuba and Moriya cities, about three times larger than the total doses to nursing mothers, although the total intake amounts of the nursing mothers were greater because of significant differences in the thyroid dose coefficients between the two age groups.

This study suggested that breast milk consumption from nursing mothers living in areas with mild I-131 pollution could be the major intake pathway for breast-fed infants. To the best of our knowledge, this work provides the first estimation of the thyroid doses to breast-fed infants consuming I-131-contaminated breast milk. However, it should be mentioned that extremely high contamination with I-131 was found in some outdoor-grown vegetables (e.g., spinach) of Ibaraki prefecture, and especially in the northern area closer to Fukushima^20^. The possibility of incidental ingestion of such contaminated foods is expected to be limited to only a few people because of damaged infrastructure and the restriction of food distribution shortly after the accident^21^; nonetheless, it would be quite important to confirm this for individual dose estimation by means of personal behavior surveys.

## Methods

### Existing measurement data

**Figure 3:**
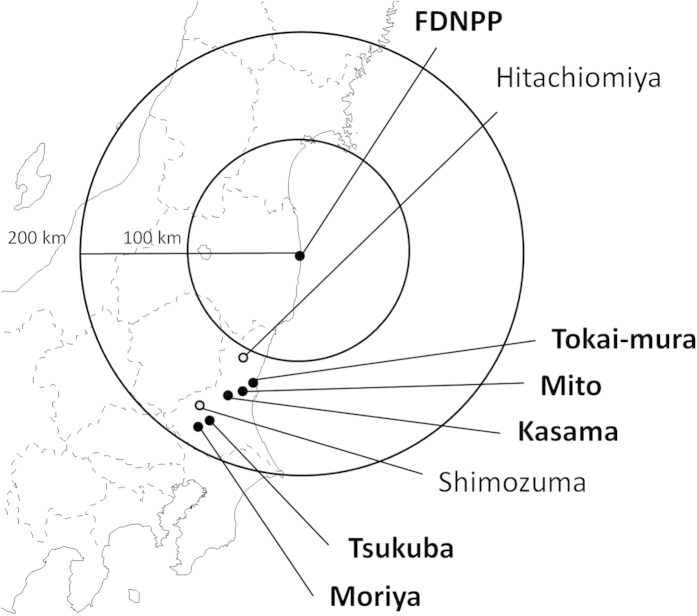
Centers of cities where Persons A-H lived including FDNPP and Tokai-mura. A copyright-free map (http://www.freemap.jp/) was used to create the figure.

Unno *et al.*^12^ published measurement data of I-131 concentration in breast milk samples collected from nursing mothers acting as volunteers. The result showed that I-131 was detected in 13 out of 60 breast milk samples in total (Table 3). The I-131 concentrations in the 13 samples ranged from 2.2–36.3 Bq/kg; however, it should be noted that these values are only a few times higher than the Detection Limits (DLs) of the measuring systems used by both groups: 1.0–7.6 Bq/kg. In addition, samples were taken only once or twice from each volunteer. The precise sampling time during the day was also unknown. In this study, the data of the six volunteers (Persons A-F) in Ibaraki prefecture (bordering Fukushima prefecture to the south) were compared by calculations described later. The centers of the cities where the six volunteers lived are located 100 to 200 kilometers from FDNPP (denoted by circles in Fig. 3). According to information from the experts in charge of measurements of breast milk samples from these six nursing mothers, the measured I-131 concentrations had counting-statistical error below 20% (1 SD), excluding the samples of Persons B (30%) and C (37%). We noticed a relatively large counting-statistical error possibly due to low I–131 concentrations in breast milk despite a long measurement time (~ 3 h). Background subtraction in spectrometry was suitably performed for all samples using background spectra obtained on the same day of each sample measurement.

### Intake scenarios for nursing mothers

The two intake pathways considered in this study are described below.

The I-131 intake activity rates for nursing mothers through inhalation, 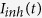, and ingestion, 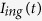, at the time of t are given as follows:


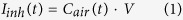



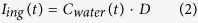


where *C*_*air*_(*t*) is the measured I-131 concentration in air at time *t* (Bq/m^3^), *C*_*water*_(*t*) is the measured I-131 concentration in tap water a*t* time *t* (Bq/kg), *V* is the daily effective breathing volume for I-131 (m^3^/d), and *D* is the daily consumption of tap water (kg/d) of the nursing mothers.

**Figure 4:**
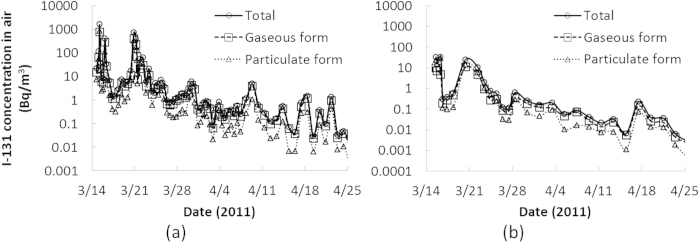
Measured I-131 concentration in air from March 14 to April 25, 2011: (**a**) Tokai-mura, (**b**) Tsukuba city.

*C*_*air*_(*t*) was obtained from air sampling data by three institutes in Ibaraki prefecture: Nuclear Fuel Cycle Engineering Laboratories of Japan Atomic Energy Agency (JAEA-NFCEL), National Institute of Environmental Studies (NIES) and High Energy Accelerator Research Organization (KEK, Japanese acronym). JAEA-NFCEL is located at Tokai-mura (Tokai village) and NIES and KEK are in Tsukuba city (Fig. 3). JAEA-NFCEL started air sampling on March 13, 2011 and NIES and KEK jointly started on March 15, 2011. The data by JAEA-NFCEL showed that the first event of the arrival of radionuclide-enriched plumes occurred on March 15. This was also reproduced by atmospheric dispersion simulations^2^,^22^. Each air sampling time by JAEA-NFCEL, NIES and KEK was 3-48 hours and most of sampling intervals were less than 15 minutes. Figure 4 displays the time series of the measured I-131 concentration in the air from 14 March to 25 April 2011^23^,^24^. For the intake scenario to be input in the biokinetic model (described later), *C*_*air*_(*t*) was assumed to be constant during each sampling period or be the same as the concentration of the last sampling period. *C*_*air*_(*t*) was also divided into two components, gaseous and particulate forms of I-131, to take into account the difference in deposition rate in the respiratory tract between the two forms. The air sampling data by JAEA-NFCEL were used for the intake scenario in Mito and Kasama cities (Persons A-C) and those by NIES and KEK were used for that in Tsukuba and Moriya cities (Persons D-F).

**Figure 5:**
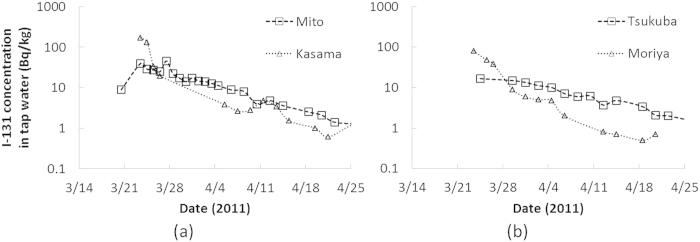
Measured I-131 concentration in tap water from March 14 to April 30, 2011: (**a**) Mito and Kasama cities, (**b**) Tsukuba and Moriya cities.

Regarding intake from contaminated water, *C*_*water*_(*t*) was obtained from tap water sampling data by Ibaraki Prefectural Environmental Radiation Monitoring Center. The water samples were collected at purification plants or general household faucets in cities within Ibaraki prefecture. Figure 5 displays the time series of the measured I-131 concentration in tap water from late March to 30 April 2011^25^. Sampling was generally performed once per day starting one week after the accident. To create the intake scenario, *C*_*water*_(*t*) on a certain day was set at zero before the sampling and was interpolated using data on the two adjacent days in the case of missing data. It was also assumed that persons consumed tap water from the nearest sampling point available: Mito city (Person A), Kasama city (Persons B and C), Tsukuba city (Persons D and E) and Moriya city (Person F). Thus, for example, the intake scenario for a nursing mother in Mito city (e.g. Person A) was created using the air sampling data by JAEA-NFCEL for inhalation and the tap water sampling data in Mito city for ingestion.

V was determined by the following equation:


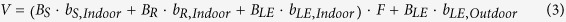


where B_S_, B_R_ and B_LE_ are the breathing volume rates at the time of sleep, rest and light exercise (m^3^/h); b_S,Indoor_, b_R,Indoor_, b_LE,Indoor_ and b_LE,Outdoor_ are the time budget values for sleep indoor, rest indoor, light exercise indoor and outdoor (h/d), and F is the reduction factor for inhalation due to staying indoor (dimensionless). Corresponding values for the breathing volume rates and time budget values for a housewife were based on published literature^15^,^26^ (Table 4). F was determined to be 0.75 based on the result of a previous study: 0.45-0.74 for the gaseous form and 0.62–0.72 for the particulate form^27^. Consequently, V was determined to be 15.2 (m^3^/d). This value can change depending on personal behavior. Considering some extreme cases, the value ranges from 13.1 (m^3^/d) for persons with no outdoor activities to 18.4 (m^3^/d) for persons with a lot of outdoor activities. Thus, variation in effective breathing volumes can result a change in calculated I-131 concentrations in breast milk only within 20%.

D was set at 0.0, 1.0 or 2.0 (kg/d), taking into account a wide range of water consumption level. No consumption was assumed for persons who might avoid drinking tap water due to concerns about radioactive contamination. In general, the daily consumption volume of tap water tends to be greater in nursing mothers than in others^28^.

### Biokinetic model for mothers during lactation

**Figure 6:**
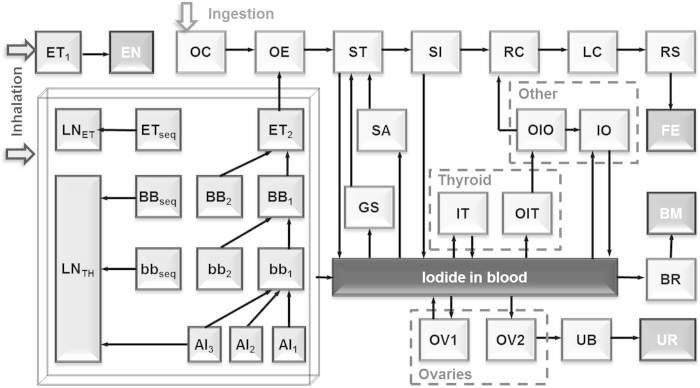
Biokinetic model for calculating I-131 concentration in breast milk. The names of the compartments are as follows: ET, extrathoracic region; EN, environment; BB, bronchial region; bb, bronchiolar region; AI, alveolar-interstitial region; LN, lymph nodes; OC, oral cavity; OE, oesophagus; ST, stomach; SI, small intestine; RC, right colon; LC, left colon; RS, rectosigmoid; FA, faeces; SA, salivary glands; GS, gastric secretory glands; IT, iodide in thyroid; OIT, organic iodide in thyroid; OIO, organic iodide in other organs and tissues; IO, iodide in other organs and tissues; OV, ovaries; UB, urinary bladder; UR, urine; BR, breasts; BM, breast milk.

The I-131 concentration in breast milk was calculated based on the biokinetic model demonstrated in Fig. 6. This model consists of the following three sub-models: the human respiratory tract model (HRTM)^15^, the human alimentary tract model (HATM)^29^ and the systemic model for iodine during lactation^14^ originally developed by Berkovski^30^. The model was numerically calculated for each exposure pathway with the Runge-Kutta method using the code by the authors^13^. The I-131 concentration in breast milk (Bq/kg) was calculated by dividing the daily total activity in the breast milk compartment by a daily secreted volume of 800 ml (=0.8 kg)^14^. Parameter values in the model, such as transfer rates between the compartments and the original regional deposition rates in the HRTM, were set to be the same as those provided in ICRP publications^15^,^16^. Regarding inhalation, the gaseous and particulate forms of I-131 were assumed to be default parameters as described in the ICRP publication 66^15^: Vapour with Class SR-1 was used for the gaseous form and Aerosols with Type F and an aerodynamic median activity diameter (AMAD) of 1 μm were used for particulate form. The intake scenario was treated in the code by separately inputting intake amounts through inhalation and ingestion for each time step of the calculations (0.001 day).

## Additional Information

**How to cite this article**: Tani, K. *et al.* Implementation of iodine biokinetic model for interpreting I-131 contamination in breast milk after the Fukushima nuclear disaster. *Sci. Rep.* **5**, 12426; doi: 10.1038/srep12426 (2015).

## Figures and Tables

**Table 1:** Comparison between measured and calculated I-131 concentrations in breast milk.

City	Person	Date of sampling	Measured value (Bq/kg)	Calculated value† **(Bq/kg)**
Mito	A	April 24	8.0	0.52–2.9
Kasama	B	April 24	2.3	0.52–2.5
	C	April 25	2.3	0.26–1.5
Tsukuba	D	March 23	8.7	19–63
	E	March 29	6.4	1.9–15
Moriya	F	March 23	31.8	19–64
	F	March 30	8.5	1.5–12

^†^The range was due to the variation of calculated concentrations on each date of sampling and that of intake scenarios.

**Table 2:** Thyroid equivalent dose to nursing mothers and their breast-fed infants (mSv).

City	Nursing mothers			Breast-fed infants		
	Inhalation		Ingestion	Inhalation		Ingestion
	G†	P†		G†	P†	
Mito	2.8 (7.2)	0.84 (5.6)	≤0.38 (≤0.86)	3.3 (1.0)	1.1 (0.78)	10–11 (2.8–3.1)
Kasama			≤0.36 (≤0.84)			10–11 (2.8–3.1)
Tsukuba	0.23 (060)	0.14 (0.92)	≤0.22 (≤0.50)	0.28 (0.080)	0.18 (0.13)	1.1–1.7 (0.29–0.45)
Moriya			≤0.26 (≤0.60)			1.1–1.8 (0.29–0.48)

Values in brackets are corresponding I-131 intake amounts (kBq).

^†^G, gaseous form; P, particulate form.

**Table 3:** Measured I-131 concentration in breast milk samples.

**Prefecture**	**City**	**Person**	**Date of sampling**	**Concentration (Bq/kg)**
Ibaraki	Mito	A	April 24, May 9	8.0, <1.2
	Kasama	B	April 24, May 8	2.3, <1.3
		C	April 25, May 8	2.3, <1.3
	Tsukuba	D†	March 23	8.7
		E†	March 29	6.4
	Moriya	F†	March 23, March 30	31.8, 8.5
	Hitachiomiya	G	April 25, May 9	3.0, <1.3
	Shimozuma	H	April 25, May 15	2.2, <1.1
Fukushima	Iwaki	I	April 25, May 8	3.5, <1.0
Chiba	Chiba	J	April 25, May 9	2.3, <1.4
	Kashiwa	K†	March 29, April 4	36.3, 14.8

^†^Data were from a Japanese citizens group; the rest were obtained by Unno *et al.* All data were from Reference 12.

**Table 4:** Breathing volume rates and time budgets to determine *V* for nursing mothers.

Category for levels for exercise	Breathing volume rate (m^3^/h)	Activity	Time budgets (h/d)
Sleep, Indoor (*b*_*S,Indoor*_)	0.32	Sleep	7.62
		Meals	1.45
Rest, Indoor (*b*_*R,Indoor*_)	0.39	Watching TV, listening to radio or reading newspapers or magazines	0.433
		Rest and relaxation	2.08
		Hobbies and amusements	0.100
Light exercise, Indoor (*b*_*LE,Indoor*_)	1.25	Personal care	1.07
		Housework	4.67
		Child care	4.37
		Other activities	0.0500
Light exercise, Outdoor (*b*_*LE,Outdoor*_)	1.25	Shopping	0.600
		Moving	0.800
		Social life	0.767

Breathing volume rates were those for Adult Females^15^ and the time budgets for non-working housewives in Ibaraki prefecture^26^.
